# Hierarchical TiO_2_ spheres as highly efficient polysulfide host for lithium-sulfur batteries

**DOI:** 10.1038/srep22990

**Published:** 2016-03-11

**Authors:** Zhi-Zheng Yang, Hui-Yuan Wang, Lun Lu, Cheng Wang, Xiao-Bin Zhong, Jin-Guo Wang, Qi-Chuan Jiang

**Affiliations:** 1 Key Laboratory of Automobile Materials of Ministry of Education & School of Materials Science and Engineering, Jilin University, Changchun 130025, China

## Abstract

Hierarchical TiO_2_ micron spheres assembled by nano-plates were prepared through a facile hydrothermal route. Chemical tuning of the TiO_2_ through hydrogen reduction (H-TiO_2_) is shown to increase oxygen-vacancy density and thereby modifies the electronic properties. H-TiO_2_ spheres with a polar surface serve as the surface-bound intermediates for strong polysulfides binding. Under the restricting and recapturing effect, the sulfur cathode could deliver a high reversible capacity of 928.1 mA h g^−1^ after 50 charge-discharge cycles at a current density of 200 mA g^−1^. The H-TiO_2_ additive developed here is practical for restricting and recapturing the polysulfide from the electrolyte.

Advanced power sources are urgently needed to meet the continuously surging demand in electric vehicles and large-scale electrochemical energy-storage systems^1^,^2^,^3^,^4^,^5^. However, current Li-ion batteries based on intercalation compound (e.g., LiCoO_2_ and LiFePO_4_) cannot satisfy this demand in terms of specific energy^6^,^7^,^8^. Li-S batteries, with a remarkably high theoretical energy density of 2567 Wh kg^−1^ and a high specific capacity of 1672 mA h^−1 ^g^−1^, have recently attracted intense interest^1^,^9^,^10^,^11^. Because of the low cost, non-toxicity and natural abundance, Li-S batteries show great promise for large-scale applications in renewable energy fields^2^,^12^. However, Li-S cells generally suffer from low sulfur utilization and poor long-term cycling stability mainly because of the dissolution of polysulfide in liquid electrolytes, which triggers a “shuttle effect” process^6^,^9^,^13^,^14^.

To solve these problems, major efforts have been centered on the development of the constraint of polysulfide dissolution^12^,^14^,^15^,^16^,^17^,^18^. More approaches, to date, have been devoted to physically confining the polysulfide within porous carbon materials such as mesoporous carbon^19^,^20^,^21^, graphene^22^,^23^,^24^ and hollow carbon spheres^20^,^25^. However, the polysulfide tends to diffuse out from the hydrophobic pores of carbon materials. This may be because carbon, being non-polar in nature, does not bind favourably with the polar and ionic sulfides^8^. Cui Yi group shown that sulfur–TiO_2_ yolk–shell composite could achieve a high specific capacity^26^. Next year, they further demonstrated that the strong chemical bonds between Ti_n_O_2n−1_ and S-species made important contribution to the improvement of the electrochemical properties^27^. Therefor, one of the targeted approaches is to bind polysulfides onto hydrophilic metal oxide.

Here, we report a strategy to effectively entrap polysulfides by a TiO_2_ host nanosheets with a polar surface. The hierarchical TiO_2_ spheres consisted of nano-plates were synthesised through a hydrothermal route. TiO_2_ spheres with a polar surface serve as the surface-bound intermediates for strong polysulfides binding (Fig. 1)^8^,^12^. After 50 cycles, it still has a capacity of 928.1 mA h g^−1^, corresponding to a capacity retention of 71%. The fine performances of S + H-TiO_2_ electrodes also demonstrated the critical role of H-TiO_2_ additive in mitigating the polysulfide dissolution into the electrolyte.

## Results and Discussion

**Figure 1:**
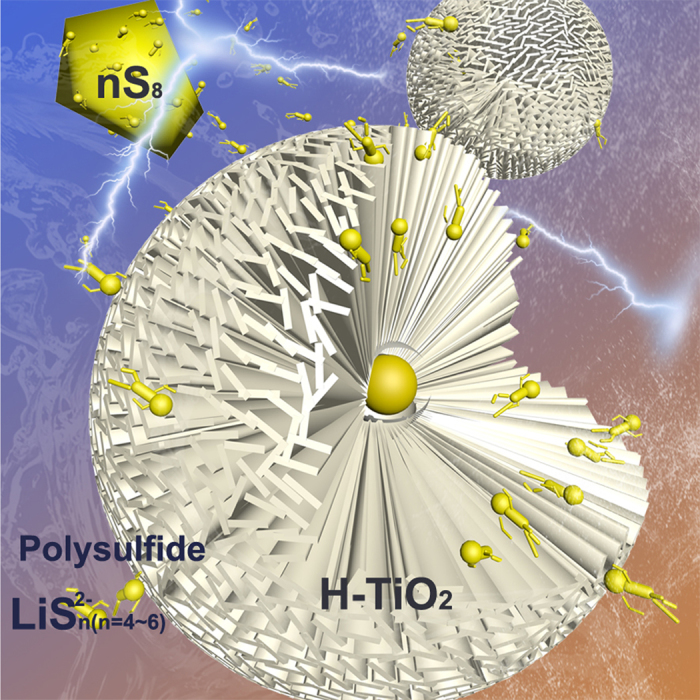
Schematic illustration of the H-TiO_2_ adsorption.

**Figure 2:**
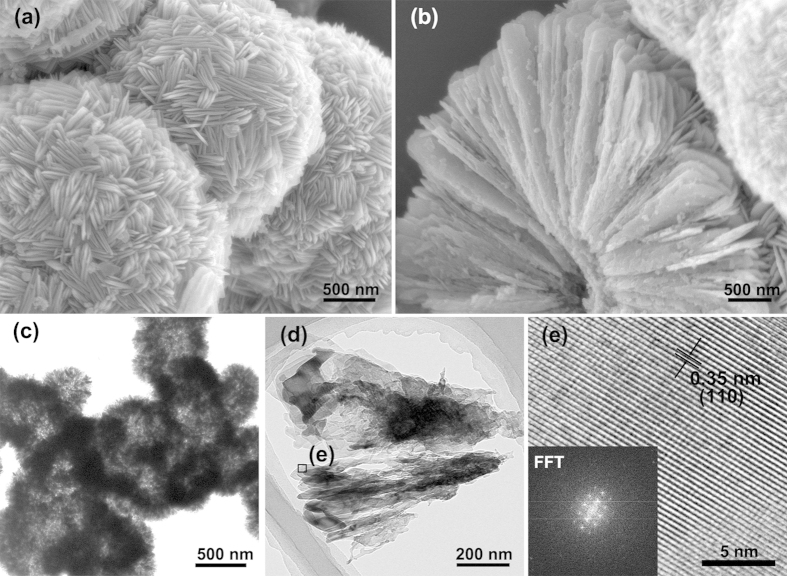
(**a**,**b**) SEM images, (**c**,**d**) TEM images and (**e**) corresponding HRTEM image of the anatase TiO_2_. Inset of (**e**) shows the corresponding Fast Fourier Transform pattern.

Figure 2a shows a typical scanning electron microscopy (SEM) image of the TiO_2_. Interestingly, the hierarchical TiO_2_ micron spheres are composed of TiO_2_ nano-plates. The nano-plates are arranged in spatial divergence form, which can be directly elucidated from the collapsed spheres (Fig. 2b). Highly ordered nano-plates are located in the core and arranged along the radial orientation to the outer surface. Hollow interior structure of as-obtained TiO_2_ spheres are also observed by TEM, as shown in Fig. 2c. Therefore, the hierarchically porous and hollow structure provide high surface area to adsorb the polysulfide dispersed in electrolyte. The TEM image of nano-plates in Fig. 1d further reveals that the component unit was fan-shaped plates with the diameter of about 70 nm and the radius of 500 nm. Fig. 2e shows the HRTEM image of an individual TiO_2_ plate with a lattice spacing of 0.35 nm, which is in good agreement with the d-spacing of (110) planes as observed in the inset of its corresponding Fast Fourier Transform pattern.

**Figure 3:**
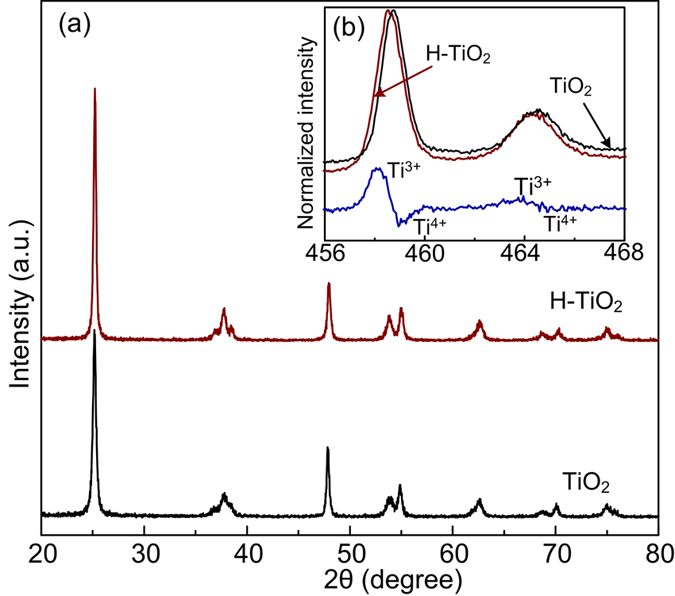
(**a**) XRD patterns and (**b**) normalized Ti 2p XPS spectra of anatase TiO_2_ and hydrogen reduced TiO_2_.

The electrical conductivity of TiO_2_ can be substantially enhanced, after annealing in a reducing gas atmosphere^9^,^28^. The anatase TiO_2_ and hydrogen reduced TiO_2_ were characterized by XRD (Fig. 3a). The identified peaks are indexed as anatase TiO_2_ (JCPDS card No. 84–1285), and no observable structural differences are observed between TiO_2_ and H-TiO_2_. There were also no obvious morphological and structure changes observed for H-TiO_2_ upon hydrogen treatment (Figure S1). To further probe the electronic and chemical environment, X-ray photoelectron spectroscopy (XPS) analysis was performed. Both spectras were calibrated with respect to the C 1s peak at 284.5 eV for clear comparison. Figure 3b (insets) illustrates the Ti 2p core level spectra for anatase TiO_2_ and H-TiO_2_. The anatase TiO_2_ sample shows two predominant peaks at 464.3 and 458.6 eV, which are attributed to the characteristic Ti 2p_1/2_ and Ti 2p_3/2_ peaks of Ti^4+^. In comparison to anatase TiO_2_, the peaks of the H−TiO_2_ sample show a red shift in binding energy, suggesting that they have different bonding environments. By subtracting the normalized Ti 2p spectra of H−TiO_2_ with TiO_2_ sample, there are two extra peaks centered at ca. 463.4 and 458.1 eV (Fig. 3b). These two peaks are within the range of published Ti^3+^ 2p binding energies^9^,^28^,^29^,^30^. The result suggests that oxygen vacancies (Ti^3+^ sites) are created in H−TiO_2_ sample during hydrogenation. It has been reported that the less positively charged Ti^3+^ nuclei leads to an increment in electron density and therefore a weaker binding effect^9^.

**Figure 4:**
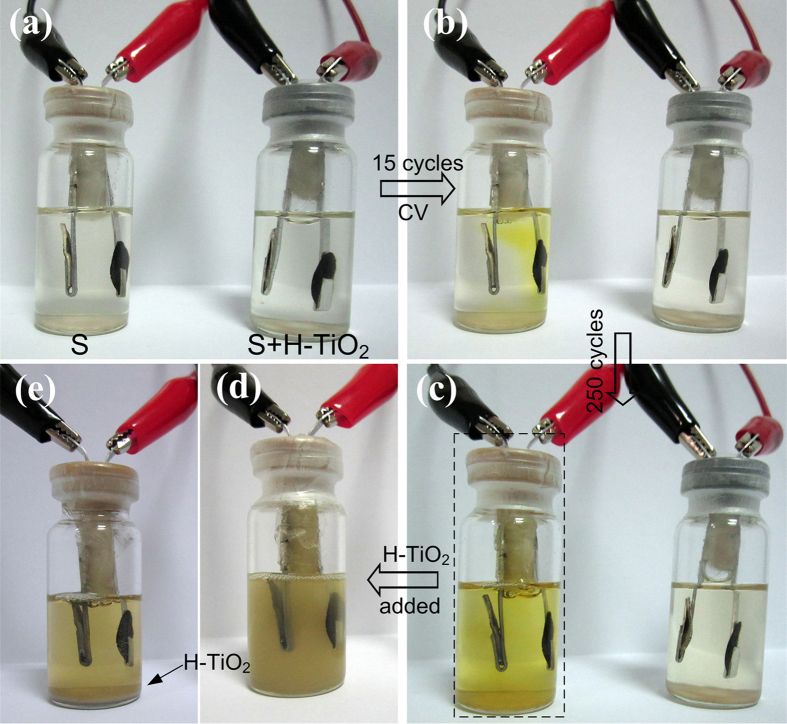
Visual confirmation of restricting and recapturing polysulfide. (**a**–**c**) Sealed vials with S and S + H-TiO_2_ cathodes under CV cycles from 1.7–3.0 V; Sealed vial with S cathode after 250 CV cycles after H-TiO_2_ added and rested for (**d**) 0 hour and (**e**) 2 hours.

Restricting and recapturing of polysulfide diffusion from additive H-TiO_2_ were clearly demonstrated through an *in-situ* visual–electrochemical study. The designed S and S + H-TiO_2_ cathodes are cycled in sealed vials against Li anodes with the same electrolyte as used in coin cells. As shown in the photograph in Fig. 4a–c, the electrolyte with S cathode changed from colourless to bright yellow during 250 cycles under the scan rate of 1 mv s^−1^. The colour change indicated that the polysulfides diffuse out of the cathode and dissolves in the electrolyte. However, the electrolyte with S + H-TiO_2_ cathode showed no obvious change, demonstrating the restriction of polysulfide diffusion from the H-TiO_2_ component. After 250 CV cycles, H-TiO_2_ was added to the bottle with pure S cathode and shook for several seconds. After resting for 2 hours, the turbid electrolyte turned clear as H-TiO_2_ precipitated, and its yellow color faded simultaneously. This demonstrates that the H-TiO_2_ additive in these cathodes also shown efficient adsorption properties for the escaped polysulfide.

**Figure 5:**
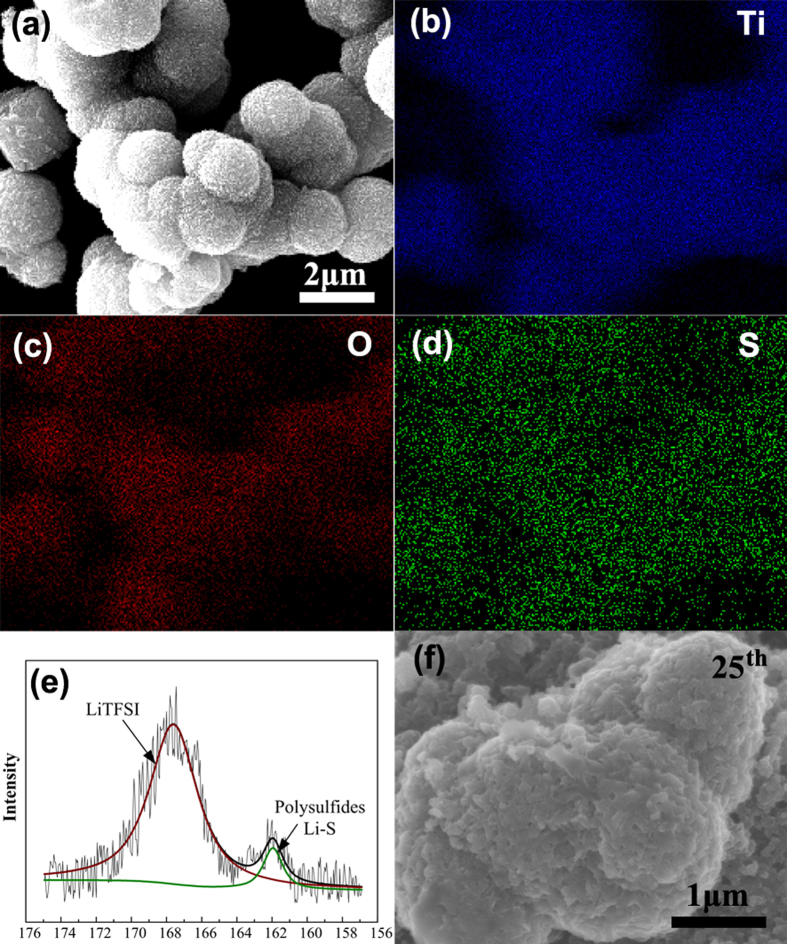
(**a**–**d**) SEM image, EDX elemental mapping of Ti, O, S, and (**e**) S 2p XPS spectra of the precipitation gathered from the sealed vial in Fig. 3e. (**e**) SEM images of S + H−TiO_2_ cathode after the 25th charge.

To further demonstrate the interaction of polysulfide and H-TiO_2_, we studied the precipitate from the sealed vial (shown as Fig. 4e). On the basis of the energy-dispersive X-ray spectroscopy (EDS) mappings (Fig. 5a–d), we conclude that element sulfur is homogeneously absorbed on the surface of H-TiO_2_. In the XPS S 2p spectra (Fig. 5e), various sulfur bonds can be observed, which also confirmed the existence of S. The peak at 166–170 eV is related to the electrolyte. As reported in previous studies, the peak in the range of 162–163 eV can be assigned to Li_2_S_x_ species^9^. Therefore, we confirmed that the H-TiO_2_ spheres were efficient in adsorption of polysulfide and deposition of S species. The S + H-TiO_2_ cathode after 25 cycles (Fig. 5f) shows little precipitation of insoluble products on the surface of the H-TiO_2_ particles.

**Figure 6:**
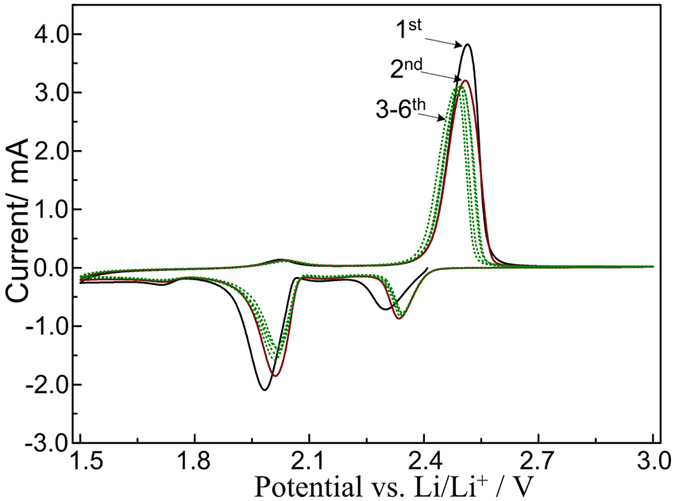
Cyclic voltammetry of the sulfur with hydrogen reduced TiO_2_ cathode obtained at 0.1 mV s^−1^.

Figure 6 depicts the typical cyclic voltammetry (CV) curves at a constant scan rate of 0.1 mV/s in the voltage range of 1.5–3.0 V. During the first cycle, two pronounced cathodic peaks at approximately 2.29 and 1.98 V are observed. According to the previously reported mechanism, the peak at 2.29 V corresponds to the transition from elemental sulfur to long-chain lithium polysulfides (Li_2_S_n_, 4 < n < 8)^6^,^9^. The peak at 1.98 V is related to the further reduction of low-order lithium polysulfides to Li_2_S_2_ and Li_2_S. The oxidation peak at about 2.52 V is associated with the reverse reactions in the charging stage. In the second CV scan, the cathodic peak at 1.98 V of the initial cathodic peak shifts to the position at about 2.01 V, and the current of the corresponding peak decreases to a low value. In the subsequent anodic cycle (3–6th cycles), the oxidation peaks shift to the position at 2.49 V, which are believed to be attributed to the complete conversion of Li_2_S into elemental S the formation of Li_2_S_n_ (n > 2)^11^,^31^. Peaks located at 1.72 V and 2.03 V reveal the lithiation and delithiation of anatase TiO_2_.

**Figure 7:**
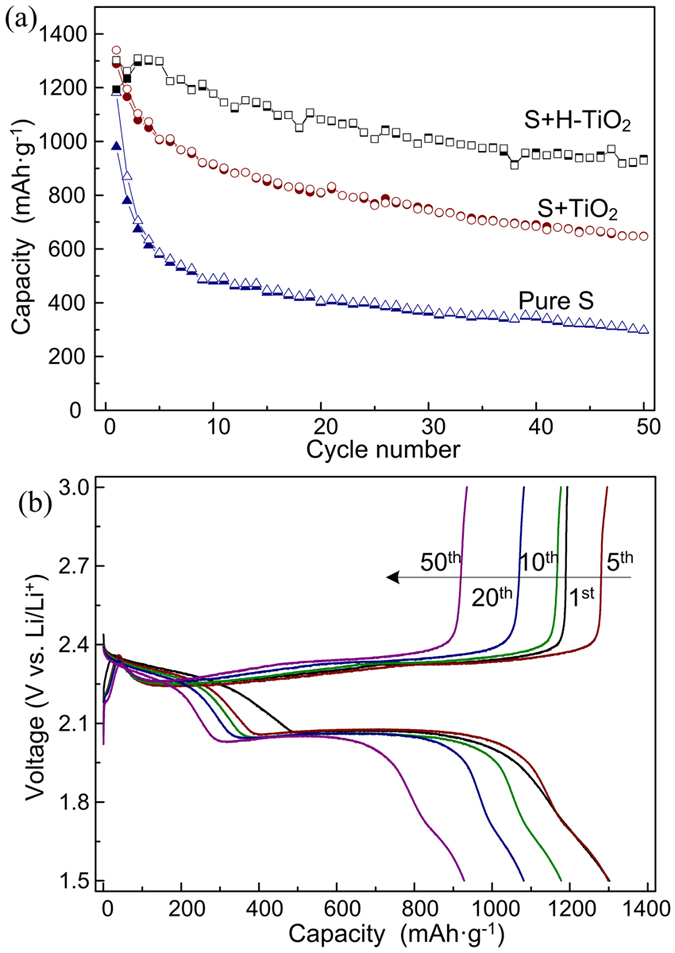
(**a**) Cycling performance of sulfur, sulfur with TiO_2_ and sulfur with hydrogen reduced TiO_2_ cathodes at 200 mA g^−1^. (**b**) Typical charge/discharge profiles of sulfur with hydrogen reduced TiO_2_ cathode at 200 mA g^−1^.

To further study the electrochemical properties, galvanostatic cycling was performed at a current density of 200 mA g^−1^. The S + TiO_2_ and S + H-TiO_2_ electrodes delivered an initial discharge capacities of 1339.4 and 1301.9 mA h g^−1^, respectively. Among them, the S + H-TiO_2_ electrode exhibited the best capacity retention, as shown in Fig. 7a. After 50 cycles, it still has a capacity of 928.1 mA h g^−1^, corresponding to a capacity retention of 71%. After 100 cycles at a current density of 1 A g^−1^, it still has a capacity of 407 mA h g^−1^ (Fig. 2s). The S + TiO_2_ electrode showed relatively poor cycling stability, but much better than that of the pure sulfur electrode. The best performance of S + H-TiO_2_ electrode also demonstrated that the critical role of H-TiO_2_ additive in mitigating the polysulfide dissolution into the electrolyte.

Figure 7b displays the charge–discharge curves of the S + H-TiO_2_ electrode cathode. Two well-defined discharge plateaus were observed, which could be assigned to the two-step reaction of sulfur with lithium. The first plateau, centered around 2.35 V, was generally attributed to the reduction of the S_8_ ring and the formation of S_8_^2−^. The discharge plateau at 2.08 V was ascribed to the further reduction of the higher polysulfides (Li_2_S_n_, 4 ≤ n ≤ 8) to the lower polysulfides (Li_2_S_n_, n ≤ 3). There exhibited two plateaus in the charge process at about 2.37 and 2.47 V, respectively. Peaks at the beginning charging profile can be attributed to H-TiO_2_. The positions of the plateaus were in accordance with the typical peaks of the S + H-TiO_2_ electrode in the CV profiles. Within the first 20 cycles, the plateaus were well preserved. In longer cycling, the capacity only dropped from 1081.3 to 928.1 mA h g^−1^, indicating that the H-TiO_2_ additive was effective in preventing the long-term loss of sulfur into the electrolyte during the redox processes. These behaviors of S + H-TiO_2_ electrode in the coin cells were consistent with what was observed in the sealed vials, where the H-TiO_2_ additive showed effective polysulfide restricting and recapturing ability.

**Figure 8:**
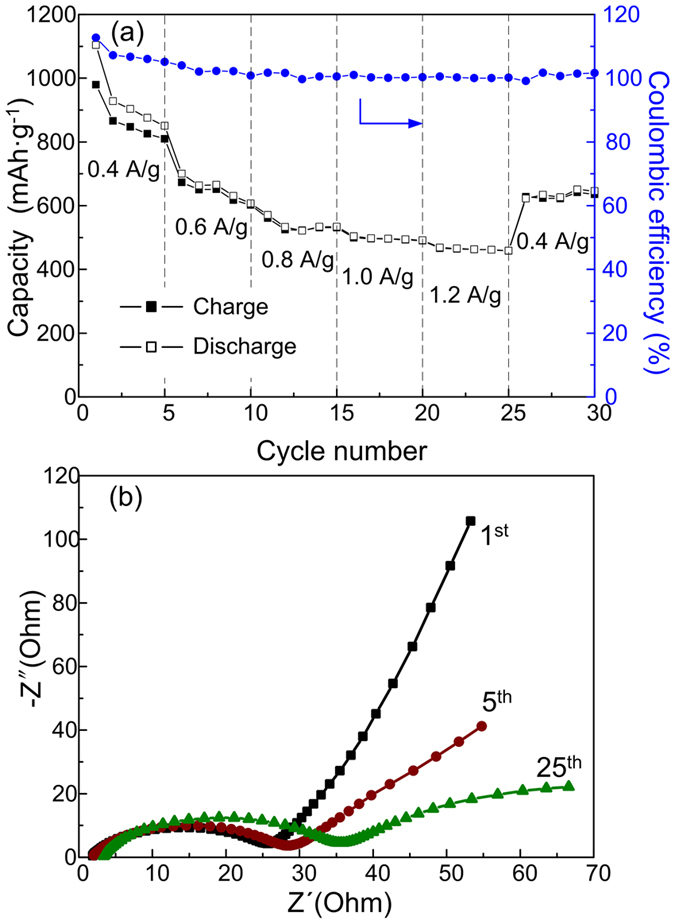
(**a**) Rate performance of the sulfur with hydrogen reduced TiO_2_ cathode at different current density from 0.4 A g^−1^ to 1.2 A g^−1^; (**b**) impedance plots of the battery at different cycles under completely charged state.

The S + H-TiO_2_ cathodes also show excellent cycling stability under continuously varying current densities, as shown in Fig. 8a. A reversible high and stable capacity of 850.8 mA h g^−1^ was retained after 5 cycles at a current rate of 0.4 A g^−1^. At a high current rate of 0.6 A g^−1^, the S + H-TiO_2_ electrode can give a reversible capacity of 650 mAh/g. When the current was further increased to 1.2 A g^−1^, a high capacity of 466.4 mAh/g can still be achieved, indicating fast reaction kinetics. It is worth mentioning that when the current was abruptly switched from 1.2 A g^−1^ to 0.4 A g^−1^ again, the capacity were recovered to 650.6 mA h g^−1^, indicating the superior robustness and reversibility of S + H-TiO_2_ electrode. The electrochemical impedance spectroscopy (EIS) was also performed to study internal resistance of the S + H-TiO_2_ and the corresponding Nyquist plots are shown in Fig. 8b. All the impedance plots for charged to 3.0 V are composed of a semicircle in the high-frequency region relating to the charge transfer resistance of the cathode and a sloping, straight line in the low-frequency region corresponding to the Li-ion diffusion within the cathode. The intersection of the semicircle on the real axis provides an approximate indication of the charge transfer resistance (Rct). This indicates that the slow accumulation of Li_2_S on the surface of S + H-TiO_2_ cathode slightly caused the increase of R_ct_ and the capacity degradation with cycle processing.

## Conclusions

Hierarchical TiO_2_ spheres consisting of nano-plates reduced through hydrogen provide polysulfides host material as a cathode additive. The reduced TiO_2_ spheres with a polar surface serve as the surface-bound intermediates for strong polysulfides binding. After 50 cycles, it still has a capacity of 928.1 mA h g^−1^, corresponding to the capacity retention of 71%. The fine performances from S + H-TiO_2_ electrodes also confirm that the critical role of H-TiO_2_ additive in restricting and recapturing the polysulfide from the electrolyte.

## Methods

### Preparation of Cathode Composites

In a typical synthesis, 1 mL of titanium tetrachloride and hydrofluoric acid were added to 40 mL of ethyl alcohol. After stirring for a few minutes, the solution was then transferred to a 50 mL Teflon-lined stainless steel autoclave and kept in an electric oven at 200 °C for 6 h. The autoclave was then taken out of the oven and left to cool naturally to room temperature. The white precipitate was harvested via centrifugation, washed thoroughly with ethanol, and dried at 60 °C overnight. Hydrogen treatment of TiO_2_ was performed by a thermal annealing process in an Ar (with H_2_ 10%) gas flow. The temperature was increased from room temperature at a rate of 2 °C /min and held at 500 °C for 3 h. The TiO_2_ or H-TiO_2_ additive (80 mg) and sulfur (160 mg) were ground together for 15 min.

### Materials Characterization

The crystalline phase of TiO_2_ and H-TiO_2_ was identified by X-ray diffraction (XRD, Dmax/2500PC, Rigaku, Japan) with Cu Kα radiation (λ = 1.5406 Å). Morphology and structure of the samples were characterized by a transmission electron microscope (TEM, JEM-2100F, 200 kV), and a field emission scanning electron microscope (SEM, JSM-6700F/Japan or FEIXL-30/USA with genesis 2000 energy-dispersive X-ray spectroscopy attachment). X-ray photo-electron spectrometry with an ESCALAB250 analyzer (XPS) were employed to characterize the obtained samples. Thermal gravimetric analysis (SDT Q600, TA Instruments Inc. USA) was carried out to estimate the amount of sulfur under Ar flow (100 mL min^−1^) at a heating rate of 10 °C min^−1^.

### Electrochemical Measurements

The TiO_2_-additive and sulfur mixtures were mixed with Super-P carbon black and PVDF binder, with a mass ratio of 60:20:20, in N-methyl-2-pyrrolidone to produce electrode slurries. The resulting slurries were then casted on carbon fiber paper (15 g m^–2^, Jiangsu China) with a laboratory doctor blade. The prepared electrode sheets were dried at 60 °C for 12 h in a vacuum oven. The typical areal density loading of active S was 0.8-1.3 mg/cm^2^. CR2025-type half-coin cells were assembled in an argon-filled glove box with the H_2_O and O_2_ contents below 1 ppm. Metallic lithium foil was used as the counter and reference electrode. Lithium bis-(trifluoromethanesulfonyl)imide (LiTFSI, 1 M) in cosolvent of 1,3-dioxolane and 1,2-dimethoxyethane (volume ratio 1:1) with lithium nitrite (LiNO_3_, 0.1 M) was used as electrolyte. Charge-discharge performances were evaluated by a LAND CT2001A battery instrument at a constant current density within a voltage window of 1.5–3.0 V at room temperature. Cyclic voltammogram measurements were carried out on an electrochemical workstation (CHI650D, Shanghai Chen Hua Instruments Ltd) at a scan rate of 0.1 mV s^−1^ from 1.5 to 3.0 V.

## Additional Information

**How to cite this article**: Yang, Z.-Z. *et al.* Hierarchical TiO_2_ spheres as highly efficient polysulfide host for lithium-sulfur batteries. *Sci. Rep.* **6**, 22990; doi: 10.1038/srep22990 (2016).

## Supplementary Material

Supplementary Information
