# Supplementary material for: Hierarchical TiO2 spheres as highly efficient polysulfide host for lithium-sulfur batteries
Source: Sci Rep. 2016 Mar 11;6:22990. doi: 10.1038/srep22990 (PMC4786857; doi:10.1038/srep22990)
Supplement: Supplementary Information [file srep22990-s1.pdf]

Hierarchical TiO_2 spheres as highly efficient polysulfide host for lithium-sulfur batteries

Zhi-Zheng Yang, Hui-Yuan Wang,* Lun Lu, Cheng Wang, Xiao-Bin Zhong, Jin-Guo Wang and Qi-Chuan Jiang

Key Laboratory of Automobile Materials of Ministry of Education & School of Materials Science and Engineering, Jilin University, Changchun 130025, China

*Corresponding author. Professor Hui-Yuan Wang (H.-Y. Wang)

E–mail address: wanghuiyuan@jlu.edu.cn (H.-Y. Wang);

KEYWORDS: titanium dioxide, lithium−sulfur batteries, sulfur cathode, host material, polysulfide

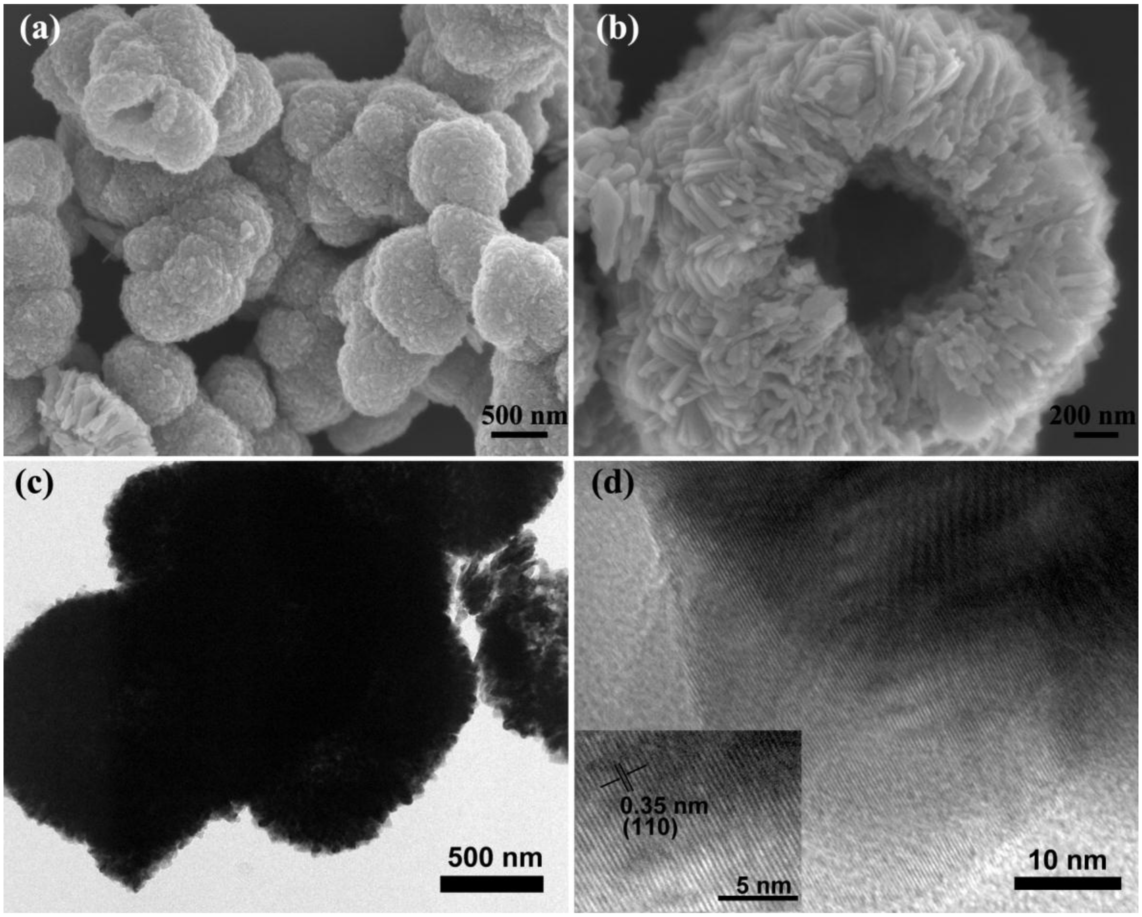

Figure s1 (a-b) SEM and (c-d) TEM images of hydrogen reduced TiO_2.

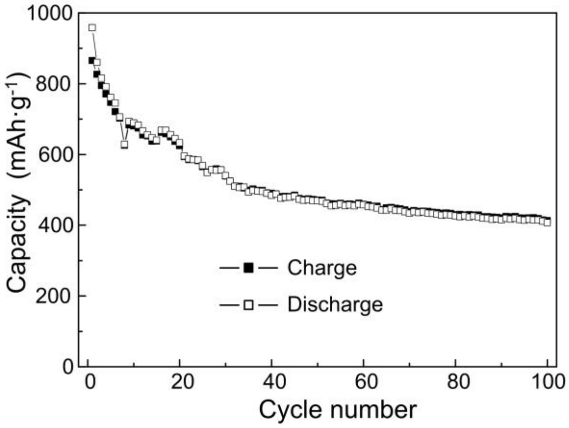

Figure s2 Cycling performance of sulfur with hydrogen reduced TiO_2 cathodes at 1 A g^{-1}.

We also tested the cycling performance again for 100 cycles at 1 A g^{-1}. After 100 cycles, it still has a capacity of 407 mA h g^{-1}.

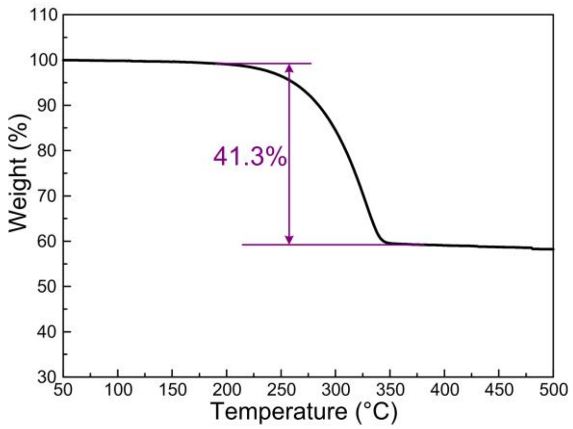

Figure s3 Thermal gravimetric analysis of H-TiO_2 /sulfur composite

During the charge-discharge cycles, active sulfur dissolved into the electrolyte, and then deposited onto the electrode surface. However, part of the polysulfide is difficult to return electrode surface, which makes the capacity declining. In this article, H-TiO_2 was shown efficient adsorption properties for the escaped polysulfide. Thus, a small quantity of fresh H-TiO_2 plays a greater role in the polysulfide recycle.

For comparison, as-prepared H-TiO_2 (100 mg) and sulfur (100 mg) were ground together, and heated to 155 ℃ in a sealed stainless steel autoclave, and kept there for 8 h to facilitate sulfur diffusion into the H-TiO_2 host. Then, the composite was heated at 200 ℃ and kept for 1 h in Ar to vaporize the sulfur deposited on the outside surface of the composite. After a thermal gravimetric analysis, it can be concluded that the saturated ratio of sulfur in this composite was 41.3%. The

proportion of sulfur with fresh H-TiO_2 was increased to 66%, which also maintained a good electrochemical property.
